# Assessing the Causal Relationship Between Various Immune Cells and Attention Deficit Hyperactivity Disorder: Mendelian Randomization Study

**DOI:** 10.1002/brb3.70280

**Published:** 2025-01-19

**Authors:** Qian Ge, Zhongyan Li, Weijing Meng, Chen Cai, Mengdi Qiu, Yafei Liu, Haibo Zhu

**Affiliations:** ^1^ Huai'an Hospital Affiliated to Yangzhou University, the Fifth People's Hospital of Huai'an Jiangsu China

**Keywords:** attention deficit hyperactivity disorder, causal inference, immune cells, Mendelian randomization, sensitivity analysis

## Abstract

**Background:**

Immune system modulation has been shown to have a significant impact on attention deficit hyperactivity disorder (ADHD). Mendelian randomization (MR) analysis was used in this study to investigate the potential role of different immune cells in the development of ADHD to provide therapy and preventative alternatives.

**Methods:**

In this study, 731 immune cells and the risk of ADHD were examined using publicly accessible genetic data and a two‐sample MR analysis. Included were four different types of immunological profiles: determinate cells (DC), proportional cells (PC), median brightness level (MBL), and morphological characteristics (MA). It was discovered that single‐nucleotide polymorphisms (SNPs) are linked to ADHD. To evaluate the dependability of the results, we conducted sensitivity analysis (heterogeneity and pleiotropy) and employed supplementary MR techniques, such as the inverse variance weighted (IVW) and MR‐Egger. Graphs are used to display the final findings of the pertinent analyses.

**Results:**

Following MR analysis, immune cells associated with a few low *p* value phenotypes may influence ADHD, and these immune cells may serve as an inspiration for clinical treatment practices that aim to prevent and cure ADHD. Immune cell phenotypes that may both increase and worsen the likelihood of having ADHD were identified by IVW results. These included CD27 on memory B cells (OR = 1.066, 95% CI = 1.024–1.109, *p* = 2E−3) and CD27 on IgD^−^CD38^−^ (OR = 1.059, 95% CI = 1.018–1.103, *p* = 5E−3), among others. Immune cell phenotypes that may act as a safeguard against ADHD included CD3 on resting Treg (OR = 0.925, 95% CI = 0.888–0.963, *p* = 1.5E−4) and SSC‐A on monocytes (OR = 0.951, 95% CI = 0.924–0.980, *p* = 8.5E−4), among others. The primary findings and the outcomes of the sensitivity analysis matched.

**Conclusions:**

This study provides a broad theoretical foundation for the development of immune‐oriented therapeutic strategies in future clinical practice by demonstrating a potential genetic relationship between immune cells and ADHD. This study also advances our understanding of how to use the immune pathway to prevent and treat ADHD.

## Graphical abstract

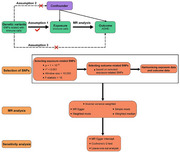

## 1 Introduction

Worldwide, children and adults suffer from attention deficit hyperactivity disorder (ADHD), a neurodevelopmental illness that is frequent (Faraone et al. 2024). Age‐inappropriate hyperactivity, impulsivity, and inattention are hallmarks of ADHD, and they all have an impact on social, academic, emotional, behavioral, and cognitive functioning (Mahase 2023). The most prevalent behavioral and neurodevelopmental condition in children is ADHD (Leung and Lemay 2003). According to reports, the condition affects 3%–11% of school‐aged children (San Mauro Martin et al. 2018; Visser et al. 2014). The treatment of ADHD has always been challenging in the current clinical practice, and the therapies used are usually multimodal, such as medication, behavioral, or educational therapies, and so forth. Given ADHD as a disorder and its complex etiology, understating the pathophysiological factors is helpful for early diagnosis and treatment. As more patients with ADHD are diagnosed, this can help control symptoms before serious complications arise, improve prognosis, and lessen the financial burden on their families.

In recent years, researchers have extensively investigated methods for treating illnesses by either stimulating or inhibiting immunological pathways. Being a crucial component of the immune response, immune cells have a significant function to play. The question of whether immune cells have a role in the onset of disease has generated a lot of attention, and research efforts have attempted to solve the puzzle. A recent study used a genome‐wide association study (GWAS) to identify gene loci linked to immune cell properties and illness risk. Numerous correlations that might serve as therapeutic targets for autoimmune illnesses were found in the study (Orru et al. 2020). The researchers discovered that immune cells are linked to a number of illnesses, such as lung cancer (Xu et al. 2024), schizophrenia (Wang et al. 2023), and generalized anxiety disorder (Ma et al. 2023), using the previously stated GWAS database. The majority of significant mental illnesses have some connection to the immune system (Hoekstra 2019). Among the psychiatric comorbidities, schizophrenia has been linked to immune cells causally. Given that ADHD is also a psychiatric disorder, research on whether ADHD is linked to immune cells will be quite meaningful. The study found that ADHD may be related to education level, but did not mention the relationship between ADHD and immune cells (Demontis et al. 2023). It has been shown that there is a high correlation between inflammation and mental health issues (Gong et al. 2023). With a risk rate ratio of more than 2, a Danish national registry cohort research demonstrated a strong association between the incidence of hospital‐associated illnesses and a higher chance of an ADHD diagnosis later (Kohler‐Forsberg et al. 2019). Particularly, the genetic risk for serum C‐reactive protein, a broad indicator of immunological activation, was linked to the genetic risk for ADHD (Hoekstra 2019). The recognition of immune system modifications in the etiology of ADHD has grown in recent years, leading scientists to delve more into the potential causative link between immunity and ADHD (Jue et al. 2024). To solve these issues, however, logical techniques of epidemiological etiological inferential analysis are required. These hurdles are brought about by small sample numbers, faulty study designs, and confounding variables that are outside the purview of current research.

**FIGURE 1:**
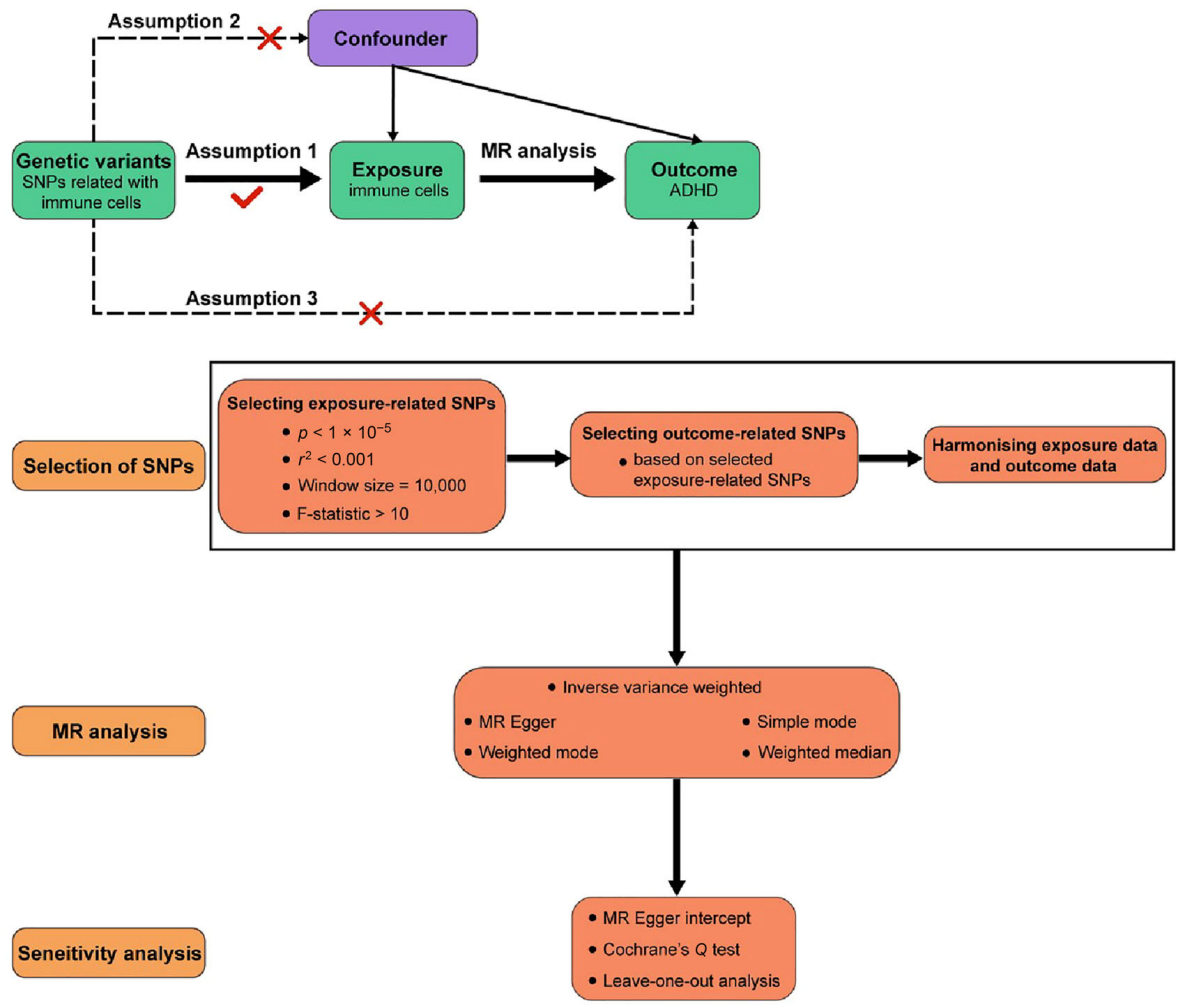
Study design and workflow of MR analysis.

Researchers have been paying close attention to Mendelian randomization (MR) analysis as a new technique for drawing conclusions about causality. The basis of MR is the Mendelian law of independent distribution, which justifies the causal order of MR by using genetic variations as independent variables to investigate causal links between exposures and outcomes (Davey Smith and Hemani 2014; Timpson, Wade, and Smith 2012). Confounders and reverse causation do not affect this impact since genetic variations are assigned at random during conception. Thus, in this investigation, we employed a two‐sample MR technique to examine the causal link between immune cell features and ADHD based on the recently released integrated information on immune cells and the combined GWAS data on ADHD. The particular procedures and related techniques for this investigation are displayed in Figure 1.

## 2 Materials and Methods

### 2.1 Study Design

Our study employed two‐sample MR analysis to examine potential causal relationships between 731 immune cell characteristics and ADHD. Three crucial MR analysis principles were adhered to when choosing the instrumental variables: (1) a clear correlation between genetic variants and exposure; (2) possible confounding effects between genetic variants and exposure and outcome; and (3) genetic variants that do not influence outcome through pathways other than exposure. Two‐sample MR analyses were conducted using a summarized dataset of large‐scale GWAS research to evaluate causal relationships between 731 immune cell characteristics and ADHD. There are no ethical concerns with any of the GWAS research included in this study because their institutions authorized them.

### 2.2 GWAS Data Sources for Exposure and Outcome

The GWAS catalog (Orru et al. 2020) has summary statistics for every immunological characteristic (GCST90001391 to GCST90002121) that are accessible to the general public. A total of 3757 Europeans who do not overlap are covered by GWAS. High‐density microarrays were utilized to interpolate genotypes for over 22 million SNPs based on a reference assembly derived from Sardinian sequencing. Following covariate correction, associations were evaluated. There were 731 immunophenotypes in all, comprising 192 relative cell counts, 32 morphological characteristics, 389 median fluorescence intensities indicating surface antigen levels, and 118 absolute cell counts.

ADHD is characterized by age‐inappropriate levels of inattention, hyperactivity, and impulsivity, all of which affect behavioral, emotional, cognitive, academic, and social functioning. The most widely used criteria for diagnosing ADHD are listed in the DSM‐5. The DSM‐5 identifies three types of ADHD: predominantly inattentive, predominantly hyperactive/impulsive, and a combined presentation (American Psychiatric Association 2013). The combined presentation is the most frequently observed subgroup, followed by the inattentive presentation and then the hyperactive/impulsive presentation (Leung 2011). The Psychiatric Genomics Consortium (PGC) provided pooled GWAS data on ADHD (ID: ieu‐a‐1183). This dataset was gathered from 20,183 individuals with ADHD and 35,191 controls across 12 cohorts (URL: https://gwas.mrcieu.ac.uk/datasets/ieu‐a‐1183/) (Demontis et al. 2019). This included a population‐based cohort consisting of 14,584 individuals with ADHD and 22,492 controls from Denmark, collected by the Lundbeck Foundation Initiative for Integrative Psychiatric Research (iPSYCH). In addition, data of 11 cohorts from Europe, North America, and China were aggregated by the PGC. Individuals with ADHD were identified through the National Psychiatric Central Research Register and diagnosed by psychiatrists at a psychiatric hospital in accordance with ICD10 (F90.0), after which they underwent genotyping using Illumina PsychChip.

### 2.3 Selection of Instrumental Variables

In this work, the causative link between several immune cell morphologies and the risk of ADHD was ascertained using single‐nucleotide polymorphisms (SNPs) as instrumental variables (IVs). We chose SNPs that, under the following circumstances, were highly connected with immune cell phenotypes based on recent research (Aru et al. 2023; Han et al. 2024). (1) The significance level was established at *p* < 1 × 10^−5^; (2) Linkage disequilibrium (LD) studies were carried out at a distance of 10,000 kb with an *r* ^2^ threshold of < 0.001; and (3) the *F* statistic was used to compute the IV strength of each immunological trait after SNPs with an *F* < 10 statistic were eliminated. Figure 1 displays the flowchart for the SNP selection.

### 2.4 Statistical Analysis

A number of MR analysis methods, including inverse variance weighting (IVW), weighted median, MR‐Egger, simple mode, and weighted mode, were employed to evaluate the causal connection between 731 immune cell morphologies and ADHD. Our main MR analysis method was the IVW method, which integrates the Wald ratios of individual SNPs using meta‐analysis. It assumes that IVs influence outcomes solely through particular exposures and provides objective causal estimates when horizontal pleiotropy is absent (Hemani et al. 2018). We regard the exposure and result as causally connected if the IVW method *p* value is less than 0.05. Furthermore, Cochran's *Q* statistic and its associated *p* value were used to evaluate the heterogeneity between the chosen independent variables. A *p* value of more than 0.05 denotes the absence of heterogeneity between the independent variables and vice versa. Naturally, some studies include variability amongst independent variables, but this is understandable and has no bearing on the validity of the findings. MR‐Egger was utilized to determine if the SNPs included in the analysis had this impact on accounting for any horizontal pleiotropy. Horizontal pleiotropy was absent if the *p* value was larger than 0.05, and vice versa. The MR analysis' presumptions are broken and the results are untrustworthy if horizontal pleiotropy is present. The study created forest plots and performed a leave‐one‐out (LOO) sensitivity analysis (Burgess et al. 2017) to evaluate the relationship between certain SNPs and the outcomes. By methodically eliminating each SNP and doing the IVW analysis again, the analysis sought to ascertain if a single SNP was responsible for the connection. Furthermore, the impact of outliers on the data was evaluated using scatter plots, and the dependability and consistency of the correlations were evaluated using funnel plots. The TwoSampleMR package (version 0.5.8) was used to conduct MR analysis in the R Language (version 4.3.2).

## 3 Results

### 3.1 Association of Different Immune Cell Phenotypes With ADHD

**FIGURE 2:**
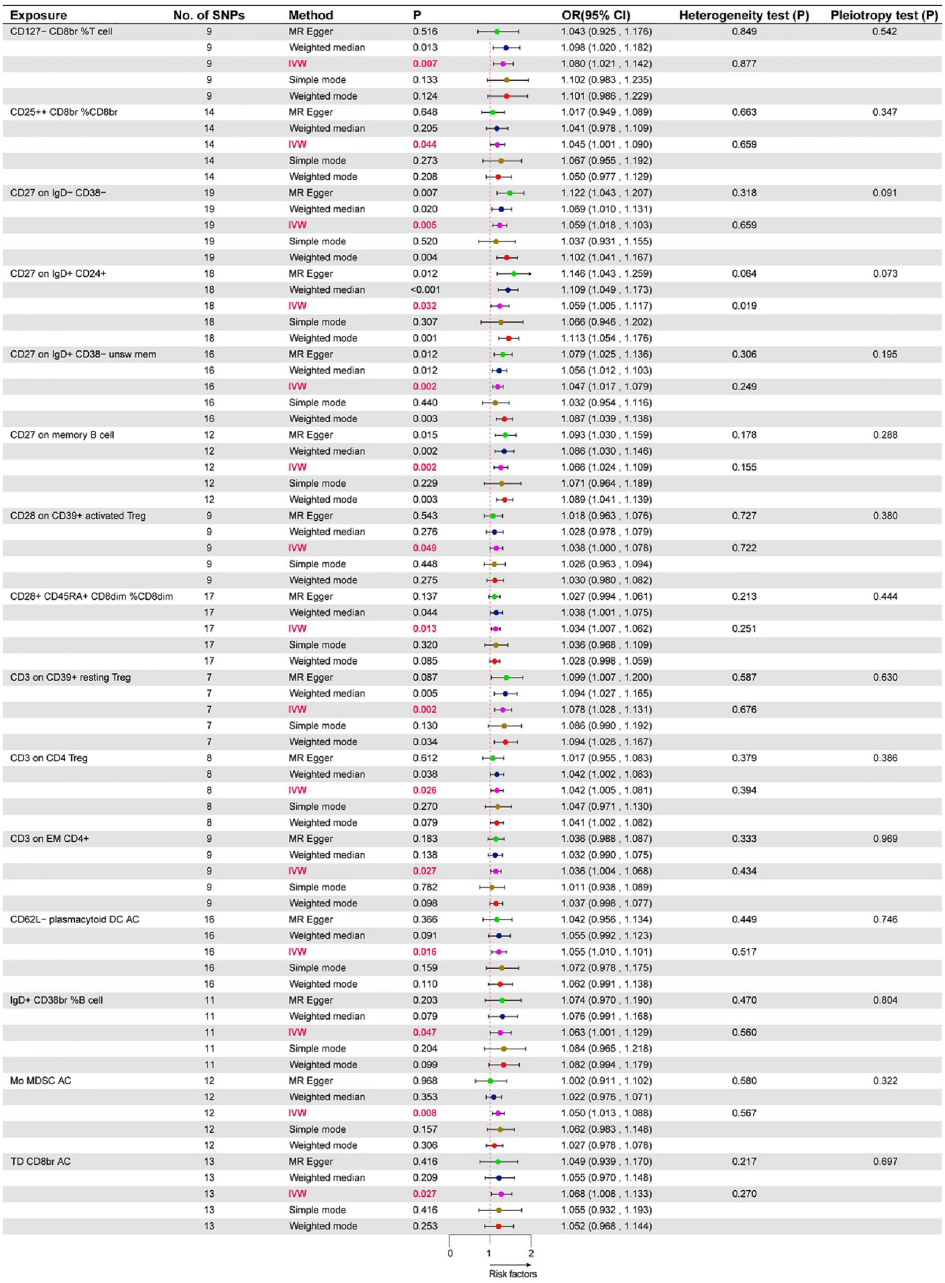
The forest map shows a causal relationship between immune traits and ADHD. The results show that 15 immune cells are risk factors for ADHD. CI, confidence interval; IVW, inverse variable weighted.

To investigate the causal relationship between immune phenotypes and the risk of ADHD, we conducted a two‐sample MR analysis, mainly using the IVW random effects model. As shown in Figure 2, under the significance threshold of *p *< 0.05 and also satisfying the condition of OR value greater than 1, we found a direct and significant association between 15 immune profiles and ADHD, which are risk factors for ADHD. MR analyses of the random effects model using the IVW approach showed that 15 immune cell traits were associated with an increased risk of ADHD. These included IgD^+^ CD38br %B cell (OR = 1.063, 95% CI: 1.001–1.129, *p* = 0.047), CD62L^−^ plasmacytoid DC AC (OR = 1.055, 95% CI: 1.01–1.101, *p* = 0.016), Mo MDSC AC (OR = 1.05, 95% CI: 1.013–1.088, *p* = 0.008), TD CD8br AC (OR = 1.068, 95% CI: 1.008–1.133, *p* = 0.027), CD28^+^ CD45RA^+^ CD8dim %CD8dim (OR = 1.034, 95% CI: 1.007–1.062, *p* = 0.013), CD25^++^ CD8br %CD8br (OR = 1.045, 95% CI: 1.001–1.09, *p* = 0.044), CD127^−^ CD8br %T cell (OR = 1.08, 95% CI: 1.021–1.142, *p* = 0.007), CD27 on IgD^+^ CD24^+^ (OR = 1.059, 95% CI: 1.005–1.117, *p *= 0.032), CD27 on IgD^+^ CD38^−^ unsw mem (OR = 1.047, 95% CI: 1.017–1.079, *p* = 0.002), CD27 on IgD− CD38− (OR = 1.059, 95% CI: 1.018–1.103, *p* = 0.005), CD27 on memory B cell (OR = 1.066, 95% CI: 1.024–1.109, *p* = 0.002), CD3 on EM CD4^+^ (OR = 1.036, 95% CI: 1.004–1.068, *p* = 0.027), CD3 on CD39^+^ resting Treg (OR = 1.078, 95% CI: 1.028–1.131, *p* = 0.002), CD3 on CD4 Treg (OR = 1.042, 95% CI: 1.005–1.081, *p* = 0.026), CD28 on CD39^+^ activated Treg (OR = 1.038, 95% CI: 1–1.078, *p* = 0.049). In addition, four other MR analysis methods (weighted median, MR‐Egger, simple mode, and weighted mode) were utilized in our study to assess the stability of the results. The results of all five analysis methods remained largely consistent (Table S1). As shown in Figure S1, the slope of the straight line in each of the result plots is greater than 0, which also proves that the 15 immune cell traits are risk factors for ADHD. Figure S2 shows the results of the forest plot of the effect of SNPs in each of the screened immune cell phenotypes on ADHD, which also confirms that the 15 immune cells are risk factors for ADHD. No pleiotropy was detected in the results, thus confirming the robustness of our findings (Figure 2).

**FIGURE 3:**
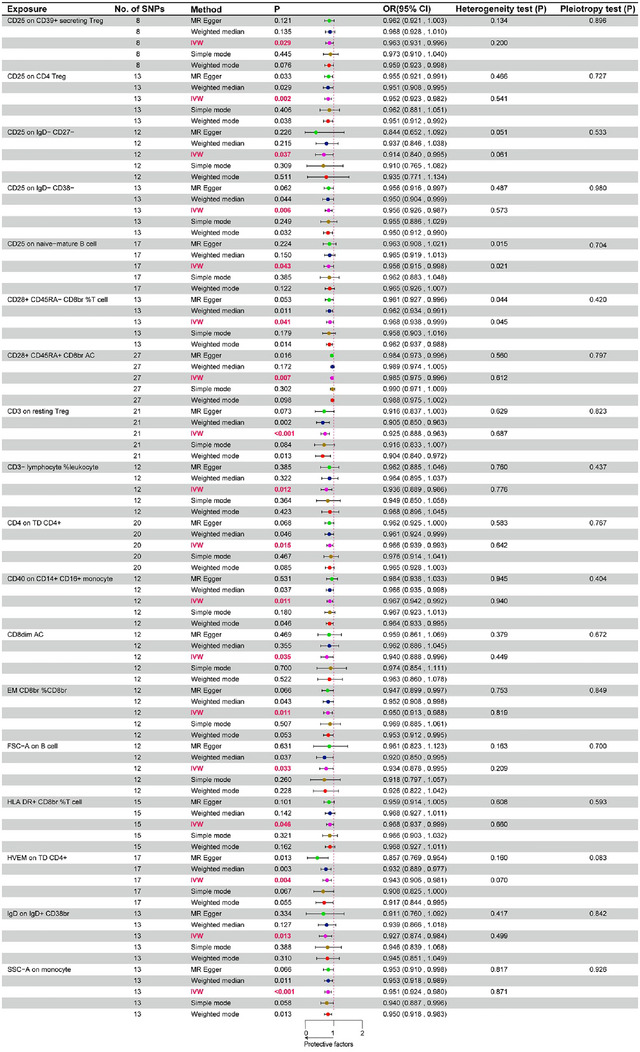
The forest map shows a causal relationship between immune traits and ADHD. The results show that 18 immune cells are protective factors for ADHD.

As shown in Figure 3, under the significance threshold of *p* < 0.05 and also meeting the condition of OR value less than 1, we found a direct and significant association between 18 immune features and ADHD, and these immune features are protective against ADHD. MR analyses of the random effects model using the IVW approach showed that 18 immune cell traits reduced the risk of ADHD. These included EM CD8br %CD8br (OR = 0.95, 95% CI: 0.913–0.988, *p* = 0.011), CD8dim AC (OR = 0.94, 95% CI: 0.888–0.996, *p* = 0.035), HLA DR^+^ CD8br %T cell (OR = 0.968, 95% CI: 0.937–0.999, *p* = 0.046), CD3− lymphocyte %leukocyte (OR = 0.936, 95% CI: 0.889–0.986, *p* = 0.012), CD28^+^ CD45RA^+^ CD8br AC (OR = 0.985, 95% CI: 0.975–0.996, *p* = 0.007), CD28^+^ CD45RA^−^ CD8br %T cell (OR = 0.968, 95% CI: 0.938–0.999, *p* = 0.041), CD25 on IgD^−^ CD27^−^ (OR = 0.914, 95% CI: 0.84–0.995, *p* = 0.037), CD25 on IgD^−^ CD38^−^ (OR = 0.956, 95% CI: 0.926–0.987, *p* = 0.006), CD25 on naive‐mature B cell (OR = 0.956, 95% CI: 0.915–0.998, *p* = 0.043), IgD on IgD^+^ CD38br (OR = 0.927, 95% CI: 0.874–0.984, *p* = 0.013), CD3 on resting Treg (OR = 0.925, 95% CI: 0.888–0.963, *p *< 0.001), HVEM on TD CD4^+^ (OR = 0.943, 95% CI: 0.906–0.981, *p* = 0.004), CD25 on CD4 Treg (OR = 0.952, 95% CI: 0.923–0.982, *p* = 0.002), CD25 on CD39^+^ secreting Treg (OR = 0.963, 95% CI: 0.931–0.996, *p* = 0.029), FSC‐A on B cell (OR = 0.934, 95% CI: 0.878–0.995, *p *= 0.033), CD40 on CD14^+^ CD16^+^ monocyte (OR = 0.967, 95% CI: 0.942–0.992, *p* = 0.011), CD4 on TD CD4^+^ (OR = 0.966, 95% CI: 0.939–0.993, *p* = 0.015), SSC‐A on monocyte (OR = 0.951, 95% CI: 0.924–0.98, *p* = 0.001). In addition, four other MR analysis methods (weighted median, MR‐Egger, simple mode, and weighted mode) were utilized in our study to assess the stability of the results. The results of all five analysis methods remained largely consistent (Table S2). As shown in Figure S5, the slopes of the straight lines in each of the result plots are less than 0, which also proves that the 18 immune cell characteristics are protective factors for ADHD. Figure S6 shows the results of the forest plot of the effect of SNPs in each of the screened immune cell phenotypes on ADHD, which also confirms that the 18 immune cells play a protective role against ADHD. No pleiotropy was detected in the findings, thus confirming the robustness of our findings (Figure 3).

### 3.2 Sensitivity Analysis

In our findings, heterogeneity was observed for three immune cell phenotypes, including CD27 on IgD^+^ CD24^+^, CD25 on naive‐mature B cell, and CD28^+^ CD45RA^−^ CD8br % T cell. *p* value exceeded 0.05 in Cochran's *Q* test for heterogeneity. We hypothesized that this could be attributed to the limited number of SNPs used as IVs. Therefore, we used a random‐effects IVW approach to examine the causal association between immune cell phenotype and ADHD. In addition, MR‐Egger intercept analysis showed that each immune phenotype did not show significant directionality or multidirectionality (*p* > 0.05), suggesting that these SNPs did not have a significant effect on the outcome through factors unrelated to exposure. No individual SNPs had an impact on the overall causal estimation, as demonstrated by LOO graphs (Figures S3 and S7), further enhancing the confidence of the MR results. In addition, the results of the funnel plot (Figures S4 and S8) showed that, except for the immune cells with heterogeneity as mentioned above, the scatters in the result plots of the other cells basically showed a symmetrical form, indicating that the results were relatively stable.

## 4 Discussion

We used a significant quantity of publicly accessible genetic data to evaluate the causal relationships between 731 immune cell characteristics and ADHD. This is the first MR study that has looked into the possible link between ADHD and various immunological phenotypes. Previous research has demonstrated that abnormalities of specific immune cell phenotypes might influence brain function and contribute to the development of ADHD; however, these studies had a number of limitations, most notably the inability to identify causality (Sreenivas et al. 2024; A. A. J. Verlaet et al. 2019; A. A. Verlaet et al. 2014; Warren et al. 1995). Unlike previous research that concentrated on single immune cell morphologies, our work provides a more thorough explanation of the causal link between several immune cell phenotypes and ADHD (Jue et al. 2024). In this study, we explored the potential causal relationship between immune cell phenotypes and the risk of ADHD through MR analyses. In doing so, we identified a series of significantly correlated immune cell phenotypes, collectively referred to as “low *p* value phenotypes.” These phenotypes showed statistical significance (*p* < 0.05) in GWAS, a result that highlights their importance in the pathogenesis of ADHD. During the screening process, we comprehensively considered the role of these immune cells in previous studies and their performance in the present study dataset to ensure the credibility and relevance of the identified phenotypes. By using an MR approach, we were able to effectively control confounding factors and thus more accurately assess the impact of these low *p* value phenotypes on ADHD risk. Our findings provide a new perspective on the clinical management of ADHD. The identified low *p* value phenotypes may serve as novel biomarkers that can help in the early identification of high‐risk individuals and drive the development of new therapies targeting ADHD. For example, by gaining a deeper understanding of the mechanism of action of these immune cells, we are expected to develop more precise preventive strategies that will not only improve treatment efficacy but also the overall quality of life of patients. Future research should focus on exploring the specific functions of these immune cells in the development of ADHD and how to translate this discovery into practical clinical interventions. We believe that this direction will not only deepen our understanding of the pathomechanisms of ADHD, but may also provide a reference for the study of other neuropsychiatric disorders.

In this study, we determined by IVW analysis that certain immune cell phenotypes may have a dual effect on the risk of ADHD, with both risk‐increasing and risk‐reducing potential. This finding reveals the complexity of the immune system in the pathogenesis of ADHD, and its clinical implications cannot be ignored. First, we observed that an increase in specific immune cell phenotypes may be associated with an elevated risk of ADHD. This result suggests that these cells may influence neurodevelopment by promoting inflammatory responses, thus providing new biomarkers for ADHD development. For example, if these immunomarkers are used as a screening tool for high‐risk individuals, they could provide a basis for early intervention and individualized treatment. On the other hand, we have also identified another class of immune cell phenotypes that may have anti‐inflammatory effects, thereby mitigating the risk of ADHD. Understanding this interaction not only helps reveal the biological basis of ADHD, but also provides a new direction for future intervention studies. Based on this finding, future studies could explore interventions targeting specific immune cells, such as modification of diet, exercise, or medication, to improve symptoms in ADHD patients. Finally, given the potential implications of this finding for public health policy, emphasizing the relationship between children's mental health and immune health will help develop more effective prevention strategies. Therefore, we recommend that future studies continue to delve deeper into the links between the immune system and mental illness, with the aim of providing new insights into the management of ADHD and other mental health problems. This study investigated the potential significance of four immunosignature categories—MFI, RC, AC, and MP in the diagnosis and treatment of ADHD. The comprehensive analysis of these immune indicators provides new perspectives for understanding the pathomechanisms of ADHD and may have far‐reaching implications for clinical management. First, changes in MFI, a quantitative indicator of the expression level of immune cell surface markers, may reflect the functional status of immune cells. In ADHD patients, abnormalities in MFI may be associated with the intensity of disease‐related inflammatory responses. This finding not only helps in early diagnosis, but also provides an important basis for individualized treatment strategies, especially when choosing anti‐inflammatory or immunomodulatory therapies. Second, RC, as an indicator of the proportion of specific immune cells in the overall immune cells, can reveal the immune characteristics of ADHD patients. By comparing the RC of ADHD patients with that of healthy controls, we were able to identify subpopulations of immune cells that are significantly increased or decreased in the disease, thus providing insight into the immune status of ADHD and its association with clinical symptoms. In addition, changes in RC may also indicate disease activity, informing the development of clinical intervention strategies. AC, on the other hand, directly describes the absolute number of specific immune cells, accurately reflecting the immune load in the patient's body. In the context of ADHD, changes in AC are closely related to disease severity, progression, and response to treatment. Therefore, regular assessment of AC can be an important indicator for monitoring changes in the condition of ADHD patients, providing real‐time feedback to the clinic, which in turn can help to adjust the treatment regimen. Finally, MP involves the morphological characteristics of immune cells, and its alteration may reflect the degree of activation or apoptotic state of the cells. By analyzing MP in ADHD patients, we may reveal the potential role of specific immune cells in neurodevelopment and thus propose new biomarkers that could further influence the future direction of treatment. In summary, the systematic analysis of the four immune profiles, MFI, RC, AC, and MP, not only deepens our understanding of the pathogenesis of ADHD, but also helps to identify high‐risk individuals at an early stage and provides a scientific basis for individualized treatment. Future studies should continue to explore how these immune features affect the clinical manifestations of ADHD to promote the development of precision medicine. Results showed that 15 immune cell phenotypes (e.g., IgD^+^ CD38br %B cell, CD62L^−^ plasmacytoid DC AC, Mo MDSC AC, TD CD8br AC, CD28^+^ CD45RA^+^ CD8dim %CD8dim) were associated with an increased risk of ADHD. In contrast, 18 immune cell morphologies exhibited protection against ADHD, including EM CD8br% CD8br, CD8dim AC, HLA DR^+^ CD8br% T cell, CD3^−^ lymphocyte%leukocyte, and so on.

Does ADHD have anything to do with immunity? The current study offers a preliminary explanation for the high correlation between immunological traits and ADHD. In addition, genetic vulnerability to tuberculosis (TB), childhood ear infections, psoriasis, rheumatoid arthritis, and serum C‐reactive protein (a broad measure of immunological activation) have all been linked to a higher genetic risk for ADHD (Hoekstra 2019). The autoantibodies of dopamine transporter proteins are related to the severity of symptoms in children with ADHD, suggesting that autoimmunity may be the cause of ADHD (Adriani et al. 2018). Because dopamine can influence the interaction between the immunological and neurological systems, the authors hypothesize that these autoantibodies may be linked to the deregulation of the neuroimmune system. Few studies have reported an association between specific immune cell phenotypes and ADHD, despite the fact that studies have shown that autoimmune disorders are associated with ADHD, either by having an immune disorder themselves or by having a parent with an immune disorder that is a risk factor for ADHD (Nielsen, Benros, and Dalsgaard 2017; Williams 2017; Instanes et al. 2017). The current study offers novel approaches for the prevention and treatment of ADHD in clinical practice, as well as strengthening the link between immunological characteristics and ADHD.

Based on published data from a sizable GWAS cohort with a sizable sample size, this study used a two‐sample MR analysis with great statistical efficiency. The study's findings were predicated on genetic instrumental factors, and many MR analytic techniques were employed to draw conclusions about causality. The outcomes were solid and unaffected by horizontal similarities with other variables. There are certain restrictions on our research. First, it was not possible to adequately evaluate horizontal commonality, even after doing several sensitivity analysis. Second, comprehensive population stratification was not possible due to the absence of specific patient characteristics in the genetic data, such as patient age and ADHD severity. Despite using a random‐effects model‐based IVW technique to reduce such variances, these variations in clinical features also contributed to the variability of the effect of different immunophenotypes on ADHD in our investigation. Third, the study only included European participants, which limits the applicability of our findings to other racial or ethnic groups. Fourth, a reverse MR analysis was not done; further research is necessary to determine the impact of ADHD on immune cell morphologies. Fourth, a false discovery rate (FDR) was not used to control for statistical bias caused by multiple comparisons; we considered finding immune cell phenotypes associated with ADHD as much as possible to give inspiration for prevention and treatment in the clinic and, therefore, lowered the criteria for selection. Even though the data were evaluated under more lax settings, which would have led to an increase in false positives, the results were significant in determining the causal relationship between immunological traits and ADHD. The next stage in this research would be to undertake a randomized controlled trial of ADHD to minimize the influence of confounding factors, increase the amount of causal evidence, and clarify processes from many angles. Finally, in this study, we explored the potential causal relationship between 731 immune cells and the risk of ADHD by two‐sample MR analysis. Our results indicate that multiple immune cell phenotypes are significantly associated with ADHD, which provides new insights into understanding the role of the immune system in the pathogenesis of ADHD. However, given the large number of immune cells involved in our study, demonstrating the association of only a single immune cell with ADHD risk is not sufficient to fully reflect its overall impact. Therefore, future studies need to consider how to integrate these immune cell data to form a more representative composite metric. For example, a polygenic risk score (PRS) could be considered, which integrates information on the genetic variation of all 731 immune cells to quantify the contribution of individual immune cells to ADHD risk. This approach can reveal the collective effect of immune cells on disease risk and help deepen our understanding of ADHD pathogenesis. In addition, load analysis is also a promising approach to assess the cumulative effect of different immune cell combinations on ADHD risk by calculating their correlations. This will help to explore the ratio of high‐risk and low‐risk immune cell cohorts, thereby further elucidating the role of immune cells in ADHD pathogenesis. In conclusion, the present study provides important preliminary evidence for the underlying immune basis of ADHD, but more in‐depth exploration is needed to fully understand the complex relationship between immune cells and ADHD risk. These efforts will not only deepen the understanding of the pathomechanisms of ADHD, but also have the potential to provide new ideas for novel therapeutic strategies for the disease.

## 5 Conclusions

In conclusion, we revealed causal connections between multiple immunological phenotypes and ADHD using MR analysis, emphasizing the immune system's complex method of action on ADHD. Immune cell phenotypes that are protective against ADHD and risk factors for ADHD were found. Furthermore, the impacts of reverse causality, unavoidable confounders, and other variables were greatly diminished by our investigation. This may give researchers a new way to investigate the underlying causes of ADHD and provide early intervention and treatment for ADHD. Our findings add to the body of psychoimmunological research and offer important new insights into the prevention of ADHD.

## Author Contributions

**Qian Ge**: writing–original draft, writing–review and editing, software, data curation. **Zhongyan Li**: software, data curation. **Weijing Meng**: investigation, validation. **Chen Cai**: methodology, visualization. **Mengdi Qiu**: data curation, software. **Yafei Liu**: methodology, software. **Haibo Zhu**: writing–review and editing, project administration, supervision, conceptualization.

## Ethics Statement

The authors have nothing to report.

## Conflicts of Interest

The authors declare no conflicts of interest.

### Peer Review

The peer review history for this article is available at https://publons.com/publon/10.1002/brb3.70280.

## Supporting information



Fig.S1. Scatter plots between 15 immune cells which are risk factors and ADHD
Fig.S2. Forest plots for association of 15 immune cell which are risk factors with ADHD
Fig.S3. Leave‐one‐out plots for the causal association between 15 immune cells which are risk factors and ADHD
Fig.S4. Funnel plots between 15 immune cells which are risk factors on ADHD
Fig.S5. Scatter plots between 15 immune cells which are protective factors and ADHD
Fig.S6. Forest plots for association of 15 immune cell which are protective factors with ADHD
Fig.S7. Leave‐one‐out plots for the causal association between 15 immune cells which are protective factors and ADHD
Fig.S8. Funnel plots between 15 immune cells which are protective factors on ADHD

Supporting Information

Supporting Information

## Data Availability

The data that support this article are available in both the paper and its online Supporting Information.
