# Supplementary material for: Assessing the Causal Relationship Between Various Immune Cells and Attention Deficit Hyperactivity Disorder: Mendelian Randomization Study
Source: Brain Behav. 2025 Jan 19;15(1):e70280. doi: 10.1002/brb3.70280 (PMC11743990; doi:10.1002/brb3.70280)
Supplement: Supplementary file 1 — Fig.S1. Scatter plots between 15 immune cells which are risk factors and ADHD Fig.S2. Forest plots for association of 15 immune cell which are risk factors with ADHD Fig.S3. Leave‐one‐out plots for the causal association between 15 immune cells which are risk factors and ADHD Fig.S4. Funnel plots between 15 immune cells which are risk factors on ADHD Fig.S5. Scatter plots between 15 immune cells which are protective factors and ADHD Fig.S6. Forest plots for association of 15 immune cell which are protective factors with ADHD Fig.S7. Leave‐one‐out plots for the causal association between 15 immune cells which are protective factors and ADHD Fig.S8. Funnel plots between 15 immune cells which are protective factors on ADHD [file BRB3-15-e70280-s001.docx]

**Supplementary materials**

Assessing the causal relationship between various immune cells and attention deficit hyperactivity disorder: Mendelian randomization study

Qian Ge, Zhongyan Li, Weijing Meng, Chen Cai, Mengdi Qiu, Yafei Liu, Haibo Zhu*

Huai’an hospital affiliated to Yangzhou university, the fifth people’s hospital of huai’an, Jiangsu, China

*CORRESPONDENCE

Haibo Zhu, email: [15861719109@163.com](mailto:15861719109@163.com)

| 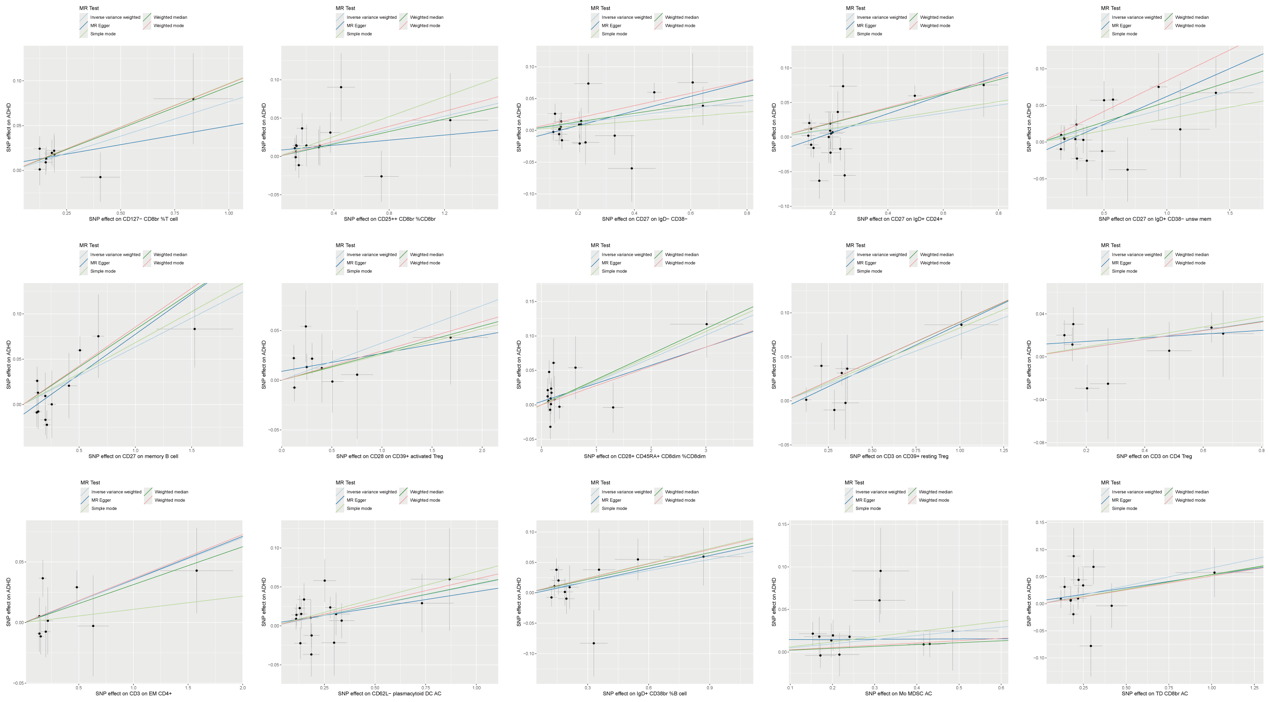 |
| --- |
| Fig.S1. Scatter plots between 15 immune cells which are risk factors and ADHD |
|  |
| 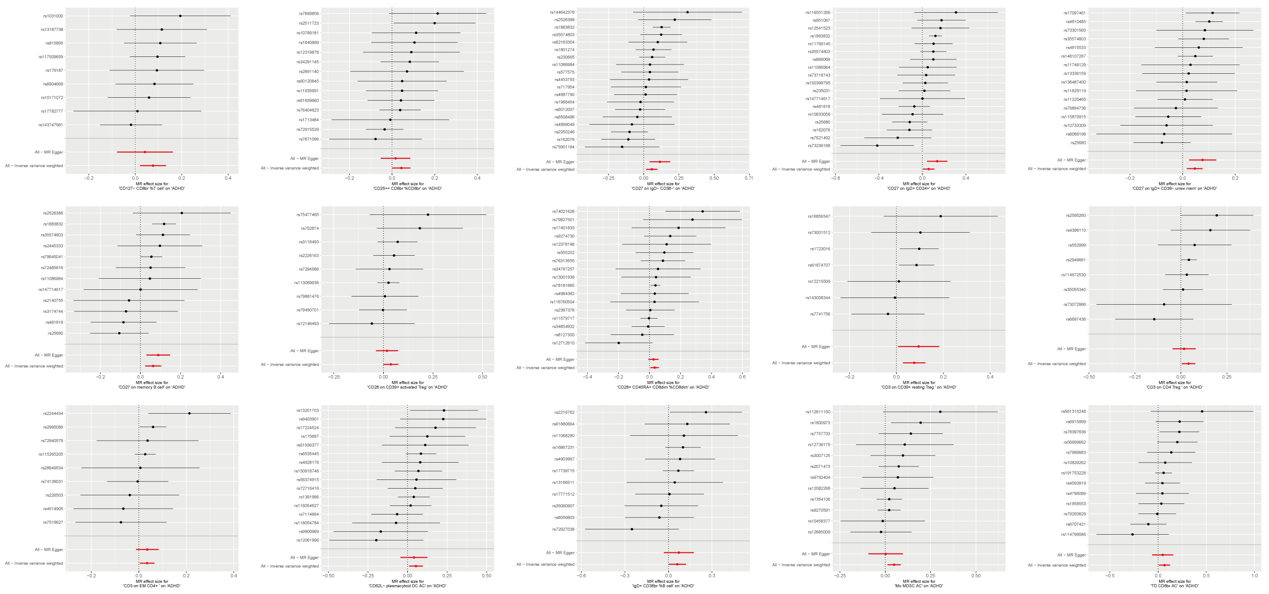 |
| Fig.S2. Forest plots for association of 15 immune cell which are risk factors with ADHD |
|  |
| 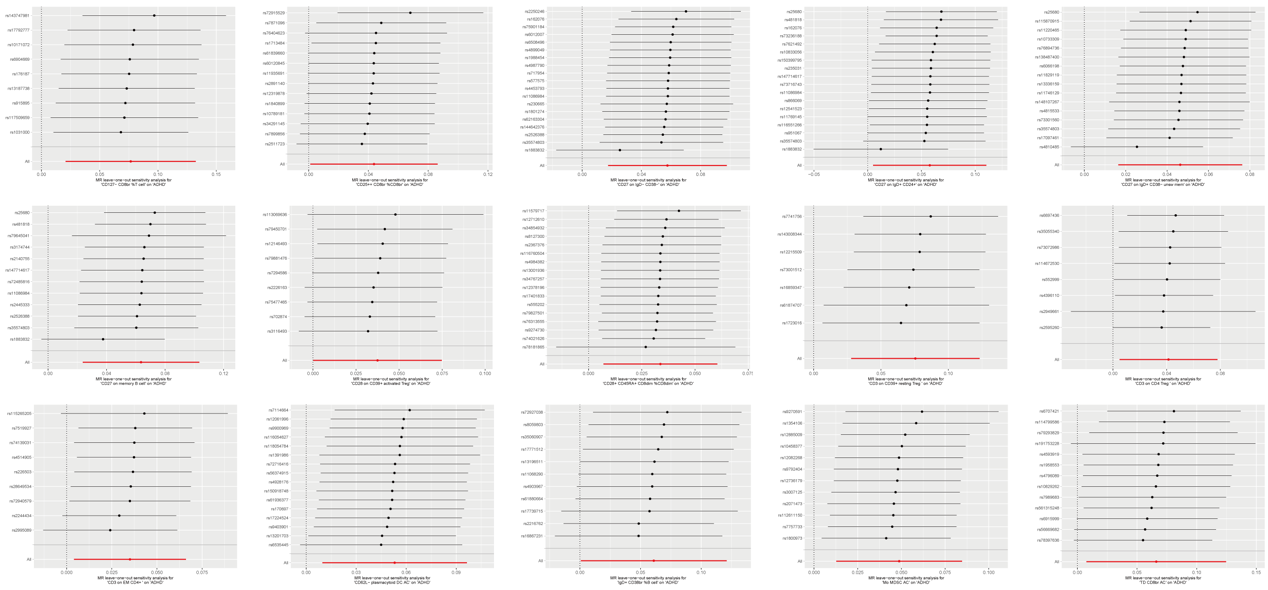 |
| Fig.S3. Leave-one-out plots for the causal association between 15 immune cells which are risk factors and ADHD |
|  |
| 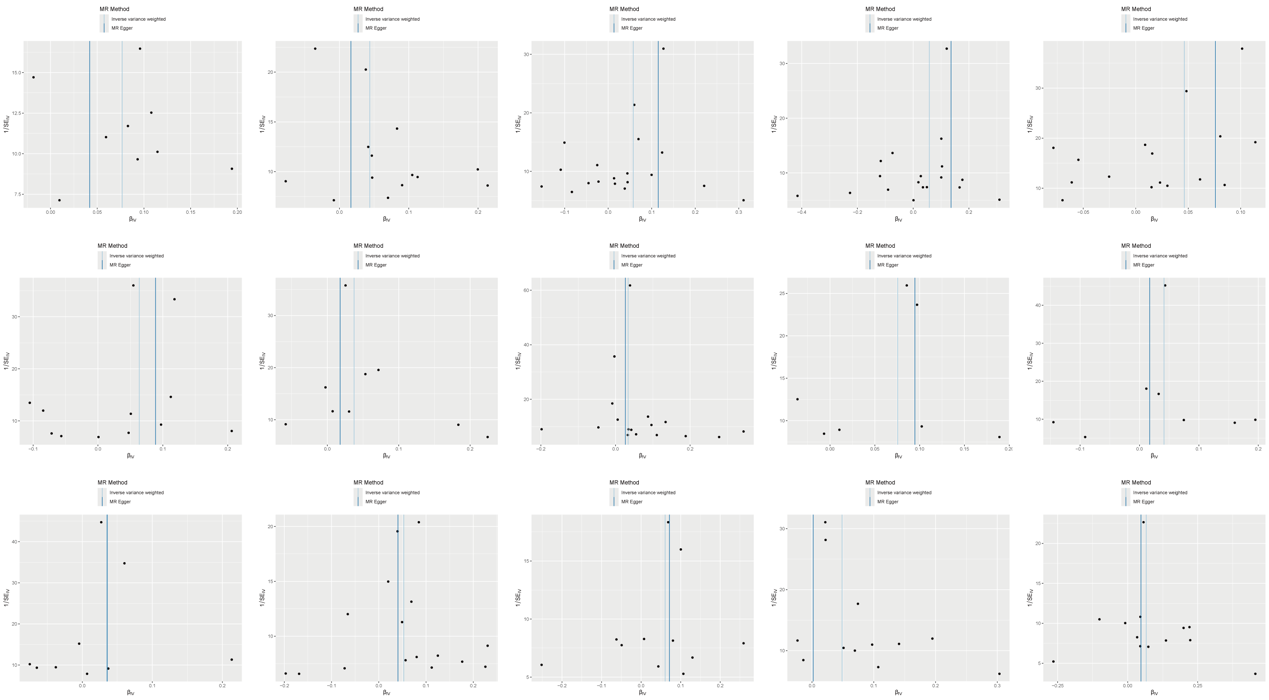 |
| Fig.S4. Funnel plots between 15 immune cells which are risk factors on ADHD |
|  |
| 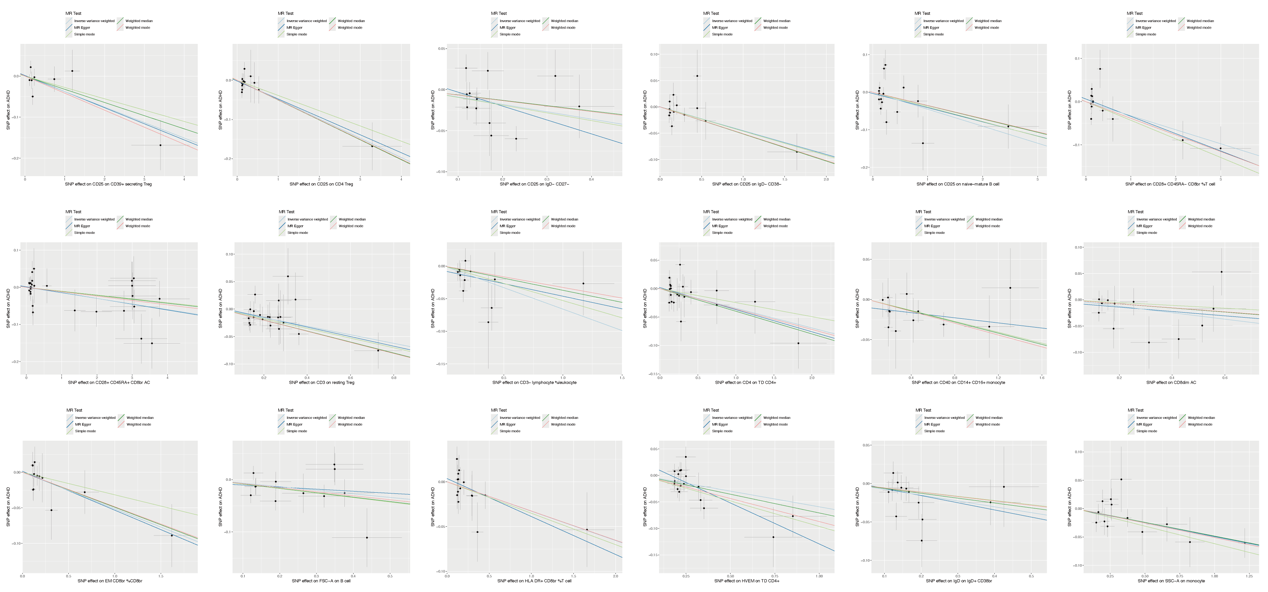 |
| Fig.S5. Scatter plots between 15 immune cells which are protective factors and ADHD |
|  |
| 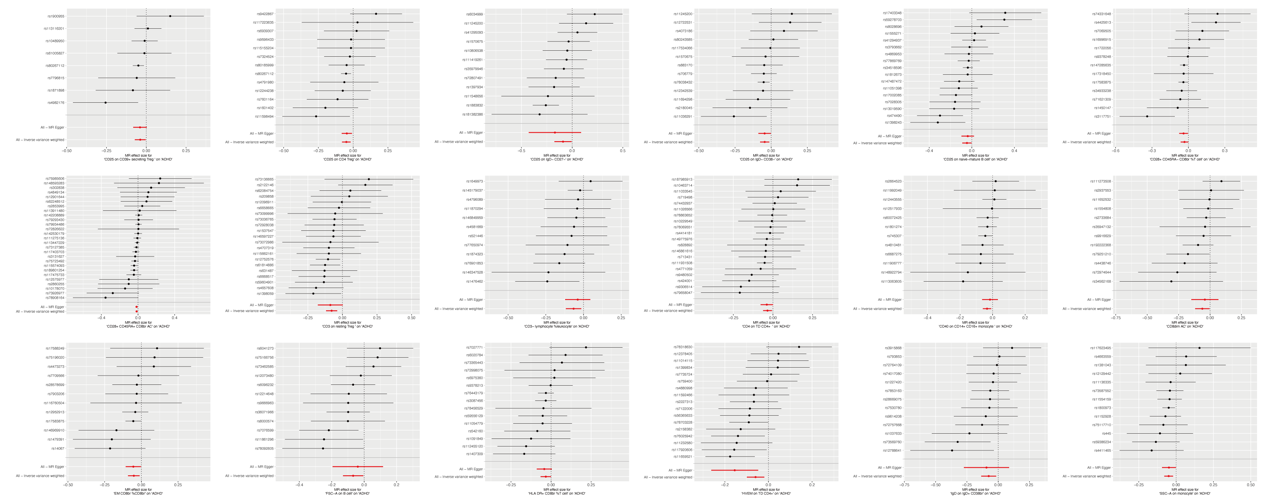 |
| Fig.S6. Forest plots for association of 15 immune cell which are protective factors with ADHD |
|  |
| 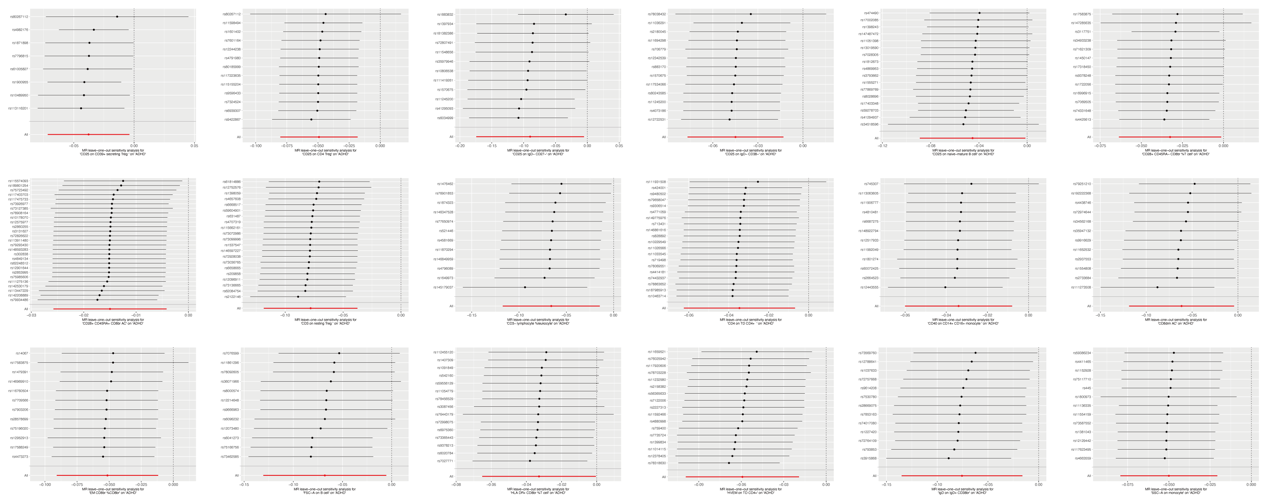 |
| Fig.S7. Leave-one-out plots for the causal association between 15 immune cells which are protective factors and ADHD |
|  |
| 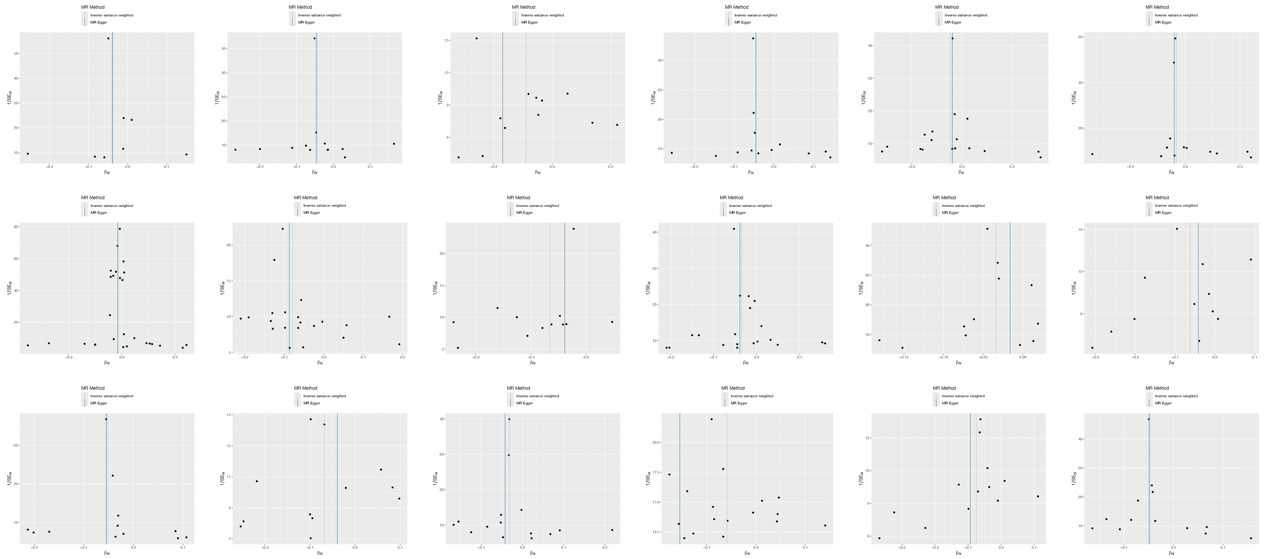 |
| Fig.S8. Funnel plots between 15 immune cells which are protective factors on ADHD |
